# Invasive meningococcal disease in patients with complement deficiencies: a case series (2008–2017)

**DOI:** 10.1186/s12879-019-4146-5

**Published:** 2019-06-14

**Authors:** Shamez N. Ladhani, Helen Campbell, Jay Lucidarme, Steve Gray, Sydel Parikh, Laura Willerton, Stephen A. Clark, Aiswarya Lekshmi, Andrew Walker, Sima Patel, Xilian Bai, Mary Ramsay, Ray Borrow

**Affiliations:** 1 Immunisation and Countermeasures Division, Public Health England, 61 Colindale Avenue, London, NW9 5EQ UK; 2 Paediatric Infectious Diseases Research Group & Vaccine Institute, Institute of Infection & Immunity, St. Georges, University of London, London, UK; 3 Meningococcal Reference Unit, Public Health England, Manchester Royal Infirmary, Manchester, UK

**Keywords:** Invasive meningococcal disease, Complement deficiency, Risk factors, Eculizumab

## Abstract

**Background:**

To describe patients with inherited and acquired complement deficiency who developed invasive meningococcal disease (IMD) in England over the last decade.

**Methods:**

Public Health England conducts enhanced surveillance of IMD in England. We retrospectively identified patients with complement deficiency who developed IMD in England during 2008–2017 and retrieved information on their clinical presentation, vaccination status, medication history, recurrence of infection and outcomes, as well as characteristics of the infecting meningococcal strain.

**Results:**

A total of 16 patients with 20 IMD episodes were identified, including four with two episodes. Six patients had inherited complement deficiencies, two had immune-mediated conditions associated with complement deficiency (glomerulonephritis and vasculitis), and eight others were on Eculizumab therapy, five for paroxysmal nocturnal haemoglobinuria and three for atypical haemolytic uraemic syndrome. Cultures were available for 7 of 11 episodes among those with inherited complement deficiencies/immune-mediated conditions and the predominant capsular group was Y (7/11), followed by B (3/11) and non-groupable (1/11) strains. Among patients receiving Eculizumab therapy, 3 of the 9 episodes were due to group B (3/9), three others were NG but genotypically group B, and one case each of groups E, W and Y.

**Conclusions:**

In England, complement deficiency is rare among IMD cases and includes inherited disorders of the late complement pathway, immune-mediated disorders associated with low complement levels and patients on Eculizumab therapy. IMD due to capsular group Y predominates in patient with inherited complement deficiency, whilst those on Eculizumab therapy develop IMD due to more diverse capsular groups including non-encapsulated strains.

## Background

*Neisseria meningitidis* (the meningococcus) remains a leading cause of bacterial meningitis and septicaemia worldwide, despite continued advances in the understanding of the pathogenesis of infection and development of new vaccines against this devastating infection. The meningococcus is commonly carried in the human nasopharynx, especially in adolescents and young adults [1, 2]. In recent years, genome-wide association studies [3] have identified host factors which may contribute to disease susceptibility; in particular, interaction between the complement system and the meningococcus has proven to be important in the pathogenesis of invasive meningococcal disease (IMD). IMD usually affects healthy individuals, in which a functional complement system acts as a first-line innate immune defence against invading pathogens [4]. Defects in components of the alternative pathway (properdin and factor D) as well as the terminal pathway (C5 to C9) underlie susceptibility to IMD [5]. Individuals with primary immunodeficiencies such as the autosomal recessive terminal complement pathway deficiencies have a 7000–10,000 fold higher risk of IMD compared to the general population and more than half of these patients develop recurrent episodes of IMD [6].

In addition to inherited deficiencies of the terminal complement pathway, a number of medical conditions and treatments can lead to acquired or secondary complement deficiency. In particular, Eculizumab (Soliris®; Alexion) is a humanised monoclonal antibody that is a terminal complement pathway inhibitor used to treat paroxysmal nocturnal haemoglobinuria (PNH) [7] and atypical haemolytic uraemic syndrome (aHUS) [8], and its use is extending to treat other immune-mediated conditions [9–13]. Eculizumab binds with high affinity to human complement C5 and blocks the generation of C5a and C5b-9, which prevents the formation of membrane attack complexes and activation of the pro-inflammatory pathway, thus protecting against end-organ damage [7].

Characterising cases of IMD in individuals with complement deficiencies is fundamental to understanding disease risk in this highly vulnerable population and developing evidence-based guidance to both prevent and rapidly treat this potentially fatal condition. Public Health England (PHE) conducts enhanced national surveillance of IMD in England and routinely follows-up all cases confirmed by its national Meningococcal Reference Unit (MRU). Here we describe age distribution, clinical presentation, risk of recurrence, meningococcal typing and outcome of IMD in individuals with inherited or acquired complement deficiency diagnosed in England over a ten-year period.

## Methods

In England, National Health Service (NHS) hospital laboratories routinely submit invasive meningococcal isolates to the PHE MRU for confirmation, grouping and additional characterisation [14]. The MRU also offers a free national PCR-testing service for patients with suspected IMD across England. IMD was defined as *Neisseria meningitidis* identified by culture or PCR from a sterile site. Confirmed cases are routinely followed up through postal questionnaires sent to their general practitioners for information on vaccination status, underlying co-morbidities, clinical presentation and outcomes of infection. For this study, IMD cases in patients with inherited or acquired complement deficiency diagnosed in England during 2008–2017 were included. Culture and non-culture samples submitted to the PHE MRU were characterised as reported previously [14–16].

## Results

A total of 16 complement-deficient patients with 20 episodes of IMD were identified during the surveillance period (Table 1). For all 20 episodes, the diagnosis was confirmed by blood culture, PCR-positive whole blood EDTA or both. Of the four cases with two IMD episodes each, the first episode occurred prior to the surveillance period in two and, for the other two, both cases occurred during the surveillance period. In three of the four patients with repeat IMD episodes, the infections were due to different capsular groups; interestingly, all three involved one episode each of group B and group W IMD. In the fourth patient, the two IMD episodes occurred more than 2 years apart and were caused by a NG(B) strain (i.e. non-groupable because of interruption of ctrA gene but genotypically capsular group B) that was confirmed by PCR only; the 2 NG(B) strains were indistinguishable in terms of PorA (P1.7–1,1) and fHbp (variant 2 peptide 23).

**Table 1:** Characteristics of patients with complement deficiency who developed invasive meningococcal disease in England

Case	Month + year of IMD	Age (years)	Detection method	Capsular group	PorA	Pen MIC	Pen	CtrA cycle number	ACWY Dose 1	ACWY Dose 2	4CMenB Dose 1	4CMenB Dose 2	Eculizumab	Underlying Condition	Antibiotic Prophylaxis
1	Jun 2011	9	Culture & PCR	Y	P1.5–1,10–1	0.125	S	20					No	Factor D	Penicillin after IMD
2a	May 2002	15	Culture & PCR	B	N	0.04	S	26	2011				No	C6 deficiency	No, rescue Amoxycillin
2b	Mar 2011	24	Culture & PCR	Y	P1.5–1,10–1	0.047	S	31							
3a	Oct 1989	20	Culture only	Y	NT^a^	0.04	S		2008				No	C8 deficiency	Amoxycillin
3b	Nov 2011	42	Culture only	B	P1.5–2,10–1	0.047	S								
4	Jun 2017	15	PCR only	NG^b^	No product	NA	NA	34					No		No
5a	Oct 2005	4	PCR only	B	ND	NA	NA	31	2016				No	C6 deficiency	No
5b	Mar 2018	16	Culture & PCR	Y	P1.5–1,10–1	0.064	S	36							
6	Mar 2017	12	PCR	Y	P1.5–2,10–2	NA	NA	30					No	C7 deficiency	No
7	Mar 2013	17	Culture only	Y	P1.5–1,10–1	0.012	S						No	Hypocomplementaemic urticarial vasculitis (anti-C1q vasculitis) on immunosuppression therapy (diagnosed in 2010)	No
8	Apr 2012	15	PCR only	Y	P1.5–1,10–1	NA	NA	28	2013		Oct 2014	Nov 2014	No	focal glomerulonephritis with persistently low complement C3/C4 (diagnosed in 2012)	Penicillin
9	Oct 2008	20	Culture & PCR	B	P1.17,16–3	0.047	S	34	Jul 2008	Dec 2011	Jul 2014	Aug 2014	Yes	PNH	Penicillin
10	Sep 2011	20	Culture only	B	P1.22,14	0.25	S		Aug 2011		Nov 2014	Jan 2015	Yes	PNH	Ciprofloxacin
11	Jan 2017	25	Culture only	Y	P1.18–1,3	0.094	S		May 2016				Yes	PNH	Penicillin
12	Oct 2009	40	Culture only	B	P1.22,14	0.064	S						Yes	PNH	No
13	Mar 2017	23	PCR only	W	P1.5,2	NA	NA	26	Dec 2016				Yes	PNH	Penicillin
14	Sep 2015	22	Culture only	NG (B) ^c^	P1.22,14	0.50	R		Mar 2015		Apr 2015	May 2015	Yes (Mar 2015)	Renal	Penicillin
15	May 2016	20	Culture only	E	P1.5,2	0.094	S		Aug 2011		Feb 2015	Apr 2015	Yes (Oct 2013)	Renal	Not known
16a	Dec 2014	20	PCR only	NG (B)^c^	P1.7–1,1	NA	NA	CtrA not detected (but siaD B CT 30)	Apr 2014		Apr 2014	May 2014	Yes (Apr 2014)	Renal	Not known
16b	Mar 2017	22	PCR only	NG (B)^c^	P1.7–1,1	NA	NA	CtrA not detected (but siaD B CT 31)							

^a^ NT. PorA phenotypically not typeable (before PorA sequencing was available); ^b^ NG. CtrA PCR positive, but negative for capsular groups A, B, C, W, & Y; ^c^ NG (B). Non-groupable because of interruption of ctrA gene but genotypically capsular group B;

*NA* Not available, a culture is needed to determine the Penicillin MICs.; *ND* Not done

Six patients had inherited complement deficiencies, and two others had immune-mediated conditions associated with complement deficiency. The age range for those with inherited complement deficiencies or immune-mediated conditions was 4 to 42 years with a median age of 15 years. The first case with an immune-mediated condition affecting complement had hypocomplementaemic urticarial vasculitis (anti-C1q vasculitis) which was first diagnosed in 2010 when the patient developed infective endocarditis needing mitral valve repair, ischaemic cerebral events, lower limb amputation and endophthalmitis resulting in complete loss of vision in one eye. This patient developed IMD 3 years later whilst on immunosuppressive therapy for the underlying condition. The second case with an immune-mediated condition affecting complement developed IMD in 2012 and was diagnosed with membranoproliferative glomerulonephritis associated with persistently low complement factors C3 and C4 four months later.

Eight other patients were on Eculizumab therapy, five for paroxysmal nocturnal haemoglobinuria (PNH) and three for atypical haemolytic uraemic syndrome (aHUS). The age range of patients on Eculizumab therapy was 20 to 40 years with a median age of 22 years. Overall, cultures were available for 7 of 11 episodes in patients with inherited complement deficiencies or immune-mediated conditions, and 6 of 9 episodes in patients receiving Eculizumab therapy despite national recommendations for daily antibiotic chemoprophylaxis in this vulnerable group. The rest of IMD episodes were confirmed by PCR only.

The predominant capsular group for the inherited complement deficiencies or immune-mediated conditions was capsular group Y (7/11), followed by capsular group B (3/11); one strain that was non-groupable by PCR (i.e. *ctrA* positive but not positive for capsular groups A, B, C, W or Y), suggesting a truly non-groupable strain or a rare capsular group. For those on Eculizumab therapy, six of the nine isolates were due to either capsular group B (*n* = 3) or non-groupable (interruption of ctrA gene) but genotypically capsular group B (NG(B); n = 3). Three other episodes were due to capsular groups W, Y and E. Capsular group Y strains was mainly associated with PorA P1.5–1,10–1. One isolate was penicillin-resistant - a non-groupable strain that was genotypically characterised as group B [17].

Four cases had recurrent IMD episodes, three who were diagnosed with an inherited complement deficiency after their second IMD episode, and one in a renal patient on Eculizumab therapy. Case 2 with complement C6 deficiency developed IMD due to capsular group B at 15 years of age, followed by IMD due to capsular group Y 9 years later. This case was on rescue antibiotics (i.e. self-treatment with a treatment course of amoxycillin when unwell) at the time and only received the MenACWY conjugate vaccine after the second episode of IMD. Case 3 with complement C8 deficiency had two episodes of IMD at 20 and 42 years of age due to capsular group Y and B, respectively; this patient was prescribed prophylactic amoxycillin twice daily following the second episode. Case 5 was diagnosed with complement C6 deficiency after the second IMD episode. This case developed IMD due to capsular group B at 4 years of age and then with capsular group Y at 16 years; the patient had received the MenACWY conjugate vaccine as part of the national UK adolescent programme 2 years prior to the second episode of IMD. Case 16 with aHUS commenced Eculizumab therapy aged 20 years and presented with IMD at 20 and 22 years of age after been appropriately vaccinated with a single dose of MenACWY conjugate vaccine and two doses of the meningococcal group B vaccine, 4CMenB. This patient was reported to be intermittently non-compliant with antibiotics. Both IMD episodes were confirmed by PCR only; the infecting strains were both PCR negative for the *ctrA* gene, but genotypically confirmed as capsular group B. Both strains had *porA* P1.7–1,1 and factor H binding protein variant 2 allele 23 peptide 23. Neither of these antigenic variants are predicted to be covered by 4CMenB.

## Discussion

We identified 16 individuals with invasive meningococcal disease who had confirmed primary or acquired complement deficiency in England over a decade. Most cases developed IMD during adolescence and early adulthood, including four who developed recurrent IMD; three of the four cases were diagnosed with an underlying primary complement deficiency after the second IMD episode. In addition to the primary complement deficiencies, we identified two groups with acquired complement deficiency, those with immune disorders leading to secondary complement deficiency and iatrogenic cases in patients receiving Eculizumab, a humanised monoclonal complement C5 inhibitor.

The complement system is an important component of the innate immune defence against invading bacteria including meningococci; complement activation on cell surfaces may be initiated through any of the three pathways - the classical, lectin, or alternative pathways. The terminal complement pathway comprises five proteins (C5-C9) that combine together to form a lethal pore-like membrane attack complex (MAC), which disrupts the bacterial cell membrane and forms transmembrane channels, leading to bacterial lysis [4]. Complement deficiencies represent approximately 1–6% of all primary immunodeficiencies but this may go up to 10% of primary immunodeficiencies in certain communities [18]. The prevalence of inherited complement deficiency has been calculated to be about 0.03% in the general European population [18], excluding mannose-binding lectin (MBL) deficiency, which has been estimated to occur in its homozygous form in about 5% of the population [19].

In our cohort, we observed an over-representation of IMD due to capsular group Y in those with inherited complement deficiencies (7/11, 64%) compared to those receiving Eculizumab therapy (1/9, 11%); the risk of group Y IMD in the latter group is similar to the rest of the population - in England, IMD due to group Y was responsible for 10.9% of all cases during 2011–15 [20]. In the UK, group Y is commonly carried in teenagers, with nasopharyngeal carriage rates of 10.0%, 10.1% and 9.7% among 15–19 year olds during 1999, 2000 and 2001, respectively [21]. During 2010–11, carriage of group Y meningococci among 18–24 year-olds in England was 7% [22].

Another significant difference between the inherited complement deficiencies and the patients on Eculizumab therapy was that all but one strain (8/9 strains, including six with group Y and two with group B) causing IMD in patients with inherited complement deficiencies were encapsulated, as were both the strains causing IMD in patients with immune-mediated conditions associated with complement deficiency (both group Y). In contrast, four of the nine IMD episodes in patients on Eculizumab therapy were either NG or group E; these strains are less virulent and usually only associated with carriage.

Our findings support the recent French report of 56 cases with terminal complement pathway deficiency who developed 61 episodes IMD between 1980 and 2015 [5]. They also found that patients with terminal complement deficiency were more likely to develop IMD due to minor or uncommon meningococcal serogroups, especially MenY which was responsible for 44% (27/61) of all cases, but also W and NG strains, although no correlation was found between specific terminal complement deficiencies and capsular group or clonal complex. Notably, as in our study, most isolates from patients with terminal complement deficiency were groupable, indicating that they were able to express capsule and could, therefore, be pathogenic [5].

A new and significant finding in our cohort was that there were more patients with IMD who were on Eculizumab treatment than those with primary terminal complement deficiency, including six treated for PNH and three for aHUS. An unwanted complication of complement inhibition, however, is an increased risk of infection with encapsulated bacteria, especially IMD. Unfortunately, just like patients with primary terminal complement deficiency, this group remains at increased risk of IMD even after appropriate immunisation because they are unable to mediate meningococcal lysis by the membrane attack complex. This finding emphasises the importance of penicillin chemoprophylaxis to prevent IMD and other serious bacterial infections in high-risk patients. Whilst there are no published guidelines on the management of individuals with inherited complement deficiencies or immune-mediated conditions associated with hypocomplementaemia, antibiotic chemoprophylaxis is routinely recommended for those with asplenia and those receiving Eculizumab. We have, however, recently reported a case of penicillin-resistant MenB disease in a vaccinated young adult on penicillin prophylaxis, highlighting the need to raise awareness of IMD risk among patients and healthcare professionals, as well as the need to seek early medical attention and for early investigation and treatment in unwell patients receiving Eculizumab, who usually present with non-specific symptoms and signs of infection. Although none of the patients in our cohort died of IMD, there have been reports of fatalities due to NG strains in patients receiving Eculizumab [23].

In addition to the known risk factors for IMD, we found other rare conditions associated with complement deficiencies, such as hypocomplementaemic urticarial vasculitis (anti-C1q vasculitis), which is associated with glomerulonephritis [24] as well as some forms of membranoproliferative glomerulonephritis. Conditions associated with acquired immune deficiencies include inadequate complement production (e.g., severe liver dysfunction), increased complement consumption (autoimmune disorders, diseases associated with immune complex formation), or increased excretion of complement components (e.g., protein-losing nephropathies) [4]. Around half the patients with systemic lupus erythematosus (SLE) have low C3 and C4 complement concentrations. C3 is the most abundant complement protein and its cleavage and the stable, covalent linkage of its fragments to target surfaces is a critical outcome of complement activation. Notably, some forms of glomerulonephritis, such as those associated with C3 nephritic factor, have responded to treatment with Eculizumab [25].

IMD in patients with complement deficiency is generally associated with mild disease, possibly due to a less intense inflammatory response in the absence of an intact complement pathway. Fatal cases have, however, been reported, including IMD cases due to NG meningococci, which generally only attack vulnerable hosts [23]. In addition to highly effective polysaccharide-conjugate vaccines against group A, C, W and Y meningococcal disease (MenACWY), there are two new broad-spectrum, protein-based meningococcal vaccines licensed in Europe. These vaccines are highly immunogenic in children and adults, and 4CMenB has been shown to protect against IMD in infants and toddlers in the field [26]. Patients with complement deficiency, however, may still develop IMD despite high post-immunisation antibody titres, again because of the lack of a functional complement pathway; vaccine failure after both MenACWY and 4CMenB have been reported in this vulnerable group, leading some specialists to recommend daily penicillin prophylaxis.

## Conclusions

Acquired and inherited deficiencies of the terminal complement pathway are rare, even in patients with IMD. Most cases of IMD in patients with inherited complement deficiency occur during adolescence. Testing for complement deficiency after a single episode of IMD is not routinely recommended because the vast majority of patients do not have any underlying co-morbidities. Healthcare professionals should, however, consider the possibility of inherited complement deficiency in any patient with recurrent IMD or in those who develop IMD due to unusual capsular groups such as Y, E or NG meningococci, especially in previously healthy adolescents. The management of such patients is challenging because of the limited protection offered by the current vaccines. In addition to vaccination, antibiotic chemoprophylaxis should be strongly considered but rare cases of penicillin-resistant group B meningococcal disease highlight the need to raise awareness of IMD risk among patients and healthcare professionals, including use of information cards to be carried by patients and their care givers, as well as the need to seek early medical attention and for early investigation and treatment in unwell patients.

## Data Availability

All data generated or analysed during this study are included in this published article [and its supplementary information files].

## Abbreviations

aHUS: Atypical haemolytic uraemic syndrome
IMD: Invasive meningococcal disease
MenACWY: Meningococcal group A, C, W and Y
MenB: Meningococcal group B
PNH: Paroxysmal nocturnal haemoglobinuria
